# SENP3-mediated deSUMOylation of Drp1 facilitates interaction with Mff to promote cell death

**DOI:** 10.1038/srep43811

**Published:** 2017-03-06

**Authors:** Chun Guo, Kevin A. Wilkinson, Ashley J. Evans, Philip P. Rubin, Jeremy M. Henley

**Affiliations:** 1 School of Biochemistry, Centre for Synaptic Plasticity, Biomedical Sciences Building, University of Bristol, University Walk, Bristol, BS8 1TD, UK

## Abstract

The GTPase dynamin-related protein 1 (Drp1) is essential for physiological and pathophysiological mitochondrial fission. DeSUMOylation of Drp1 by the enzyme SENP3 promotes cell death during reperfusion after ischaemia by enhancing Drp1 partitioning to the mitochondrial outer membrane (MOM), which causes cytochrome *c* release and apoptosis. However, how deSUMOylation recruits Drp1 to the MOM is unknown. Here we show that deSUMOylation selectively promotes Drp1 binding to the MOM resident adaptor protein mitochondrial fission factor (Mff). Consistent with this, preventing Drp1 SUMOylation by mutating the SUMO acceptor sites enhances binding to Mff. Conversely, increasing Drp1 SUMOylation by knocking down SENP3 reduces both Drp1 binding to Mff and stress-induced cytochrome *c* release. Directly tethering Drp1 to the MOM bypasses the need for Mff to evoke cytochrome *c* release, and occludes the effect of SENP3 overexpression. Thus, Drp1 deSUMOylation promotes cell death by enhancing Mff-mediated mitochondrial recruitment. These data provide a mechanistic explanation for how the SUMOylation status of Drp1 acts as a key switch in cell death/survival decisions following extreme cell stress.

Dynamin-related protein 1 (Drp1) is an oligomeric GTPase required for mitochondrial fission in mammalian cells. A key step in Drp1-mediated fission is translocation of Drp1 from the cytosol to the mitochondrial outer membrane (MOM). In response to cell stressors such as ischaemia, Drp1 partitioning to MOM plays a key role in initiating cell death^1^ and preventing Drp1-mediated mitochondrial fission can be potently cytoprotective^2^,^3^. How mitochondrial recruitment of Drp1 is regulated under physiological and pathophysiological conditions is a key unanswered question.

Drp1 comprises four main domains, a GTPase domain, a middle domain, a highly variable region (VR), and a GTPase-effector domain (GED). The VR gives rise to multiple Drp1 isoforms across species. Unlike other dynamins, Drp1 does not have a pleckstrin homology (PH) domain to interact with membrane lipids^4^ and an emerging consensus is that Drp1 is recruited to the MOM via binding of a region within the VR to specific mitochondrial docking proteins including Mff, MID49, MID51 and possibly Fis1^5^,^6^ (but see ref. 7). Drp1 binding to Mff is the predominant mechanism that promotes fission^8^,^9^ although MiD49 and MiD51 can act as alternative receptors^10^,^11^,^12^. The role of Drp1 binding to Fis1 is less clear, but it has been suggested to play an ancillary role in fission and be an important mediator of mitophagy^1^,^13^.

Protein SUMOylation is the covalent attachment **s**mall **U**biquitin-related **Mo**difier (SUMO), to lysine residues in target proteins. There are three main SUMO paralogues, SUMO-1, SUMO-2 and SUMO-3, but since SUMO-2 and SUMO-3 differ by only 3 amino acids and are usually referred to as SUMO-2/3. SUMO-1 shares 50% homology with SUMO-2/3. In general, SUMOylation acts as a biochemical switch that can regulate the function of hundreds of different proteins in multiple cellular pathways. The molecular consequences of SUMOylation are extremely diverse but one key role is the modulation of cytoprotective responses to cell stress^14^,^15^.

Drp1 can be SUMOylated by both SUMO-1 and SUMO-2/3. SUMO conjugation occurs at one or more of two pairs of lysines clustered within the VR^16^,^17^. SUMO-1-ylation of Drp1 has been reported to promote Drp1 association with the MOM^18^ whereas SUMO-2/3-ylation of Drp1 reduces binding to the MOM^17^. Thus, Drp1 modification by the different SUMO paralogues appears to have contrasting effects on Drp1 mitochondrial partitioning that results in markedly different downstream consequences^18^,^19^,^20^.

We have shown previously that SUMO-2/3 is removed from Drp1 by the SUMO protease SENP3. Importantly, SENP3 is degraded during oxygen and glucose deprivation (OGD), an *in vitro* model of ischaemia. This prolongs Drp1 SUMO-2/3-ylation and reduces Drp1 mitochondrial localization, mitochondrial fission and Drp1-dependent cytochrome *c* release. However, during reoxygenation after ischemic insult, SENP3 levels rapidly recover, leading to Drp1 deSUMOylation that promotes Drp1 mitochondrial localisation, mitochondrial fragmentation, cytochrome *c* release, and eventual cell death^17^ (see also refs 21, 22).

Here we have examined the mechanism underlying SENP3-induced mitochondrial recruitment of Drp1. We show that enhanced binding of deSUMOylated Drp1 to Mff underlies SENP3-mediated recruitment of Drp1 to the MOM and the consequent cytochrome *c* release and cell death during reoxygenation after ischaemic insult. These results define a novel molecular mechanism by which SUMOylation of a single target protein mediates cell fate decisions in response to ischemic stress.

## Results

### DeSUMOylation selectively enhances Drp1 binding to Mff

**Figure 1:**
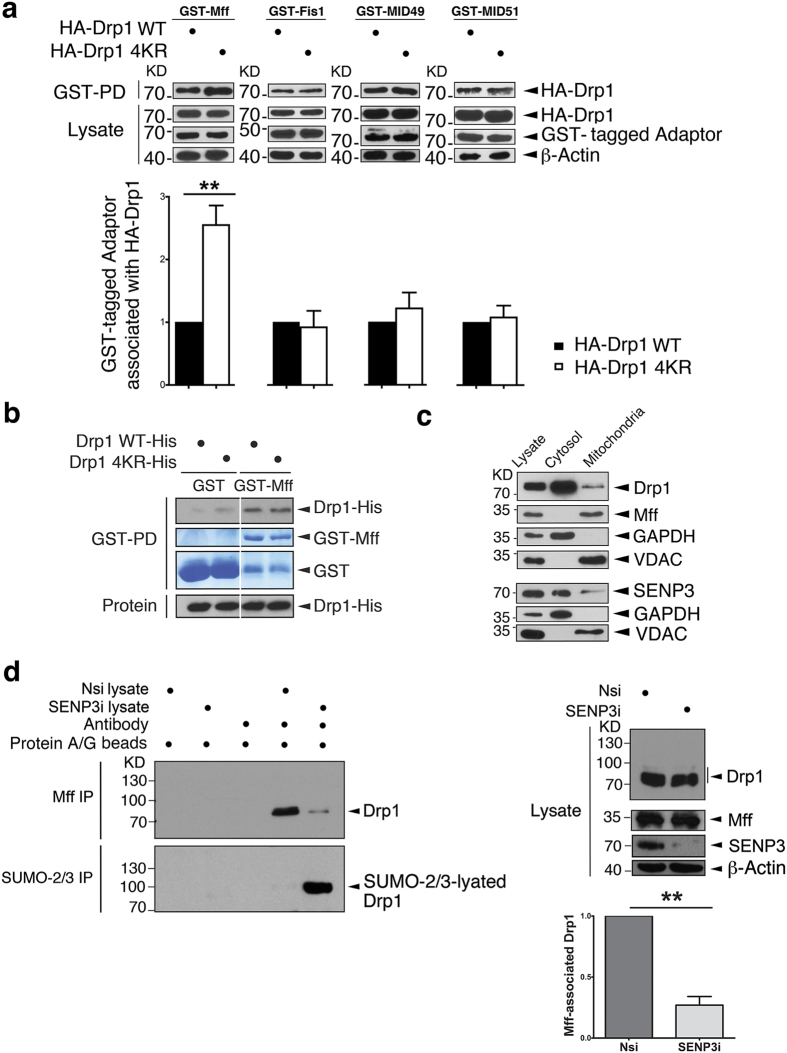
Drp1 SUMOylation selectively reduces binding to Mff. (**a**) Non-SUMOylatable HA-Drp1 shows enhanced association with GST-Mff. HA-Drp1 or HA-Drp1 4KR were transfected into HEK293 cells expressing GST-Mff, GST-Fis1, GST-MID49 or GST-MID51. GST-pull downs and lysates were immunoblotted with HA (for Drp1) and GST antibodies. The histogram shows ratio of Drp1 4KR to Drp1 binding to each receptor (For GST-Mff, n = 6; **P = 0.0042; for GST-Fis1, n = 5; P = 0.7749; For GST-MID49, n = 5; P = 0.4316; for GST-MID51, n = 5; P = 0.6926; Paired Student’s test). (**b**) *In vitro*, in the absence of SUMO, non-SUMOylatable Drp1-His and wild type Drp1-His show similar binding ability to GST-Mff. GST-PD was performed following incubation of His-tagged Drp1 or its 4KR mutant with either recombinant GST or GST-Mff. GST-pull downs and purified proteins were separated on different lanes in the same gels and immunoblotted with a Drp1 antibody. PVDF was stained with coomassie blue for GST or GST-Mff. (**c**) Cytosolic and mitochondrial localisations of Drp1, Mff and SENP3 in HEK293 cells. Drp1 and SENP3 are predominantly cytoplasmic under basal conditions and Mff is exclusively mitochondrial. GAPDH is a cytosolic marker and VDAC is a mitochondrial marker. (**d**) SENP3 knockdown, which promotes Drp1 SUMOylation, decreases the interaction between endogenous Drp1 and Mff. SENP3 siRNA (SENP3i) or non-specific siRNA (Nsi) was transfected in HEK293 cells followed by IP of Mff and SUMO-2/3. β-actin was used as a loading control. The histogram shows the degree of inhibition of Drp1 binding to Mff (n = 3; **P = 0.0091; Paired Student’s test).

We first tested how SUMOylation affects Drp1 binding its four known MOM docking proteins (Mff, Fis1, MID49 and MID51)^8^,^9^,^10^,^11^,^12^. As expected, all four proteins coimmunoprecipitate from HEK293 cells with either wild-type Drp1 or non-SUMOylatable Drp1 4KR (Fig. 1a and Supplementary Fig. 1a and b). Importantly, however, while there was no significant difference between the binding of Drp1 WT and Drp1 4KR to Fis1, MID49 or MID51, non-SUMOylatable Drp1 4KR bound ~2.5-fold more effectively than Drp1 WT to Mff. Since the lysines modified by SUMO might be within the Mff binding site we next determined if the increased binding between Drp1 4KR and Mff could be a result of the lysine to arginine mutations rather than to SUMOylation *per se*. To exclude this possibility we directly tested the binding of Drp1 WT and Drp1 4KR to Mff using bacterially purified proteins *in vitro*. We reasoned that, in the absence of SUMOylation of Drp1 WT, it should bind as well as Drp1 4KR to Mff. As predicted, there was no difference between in Drp1 WT and Drp1 4KR binding to Mff in this *in vitro* assay (Fig. 1b). These data are consistent with the hypothesis that SUMOylation of Drp1 reduces mitochondrial association by decreasing binding to Mff.

### SENP3 facilitates mitochondrial recruitment of Drp1 via Mff

We next determined the mitochondrial to cytosolic distributions of Drp1, Mff and SENP3 in HEK293 cells using subcellular fractionation. Substantial levels of SENP3 and Drp1 are present in both cytosolic and mitochondrial fractions, whereas Mff is restricted to the mitochondrial fraction (Fig. 1c). The N terminal deletion mutant of Mff (Mff∆N50) lacks the ability to recruit Drp1 to the MOM^9^. In assays using GST pull down or GFP-Trap we confirmed that Mff-WT, but not Mff∆N50, efficiently pulls down endogenous Drp1 from HEK293 cells (Supplementary Fig. 1c and d). We then manipulated levels of SENP3 and measured Drp1 binding to Mff. As expected, SENP3 knockdown significantly enhanced Drp1 SUMO-2/3-lyation and reduced the level of Drp1 associated with Mff (Fig. 1d) whereas SENP3 overexpression increased Drp1 binding to GST-Mff in pulldown assays (Supplementary Fig. 1e). Importantly, overexpression of GFP-SENP3 had no effect on the binding of the non-SUMOylatable mutant HA-Drp1 4KR to GST-Mff (Supplementary Fig. 2), suggesting that SENP3 facilitation of Drp1 binding to Mff occurs via SENP3-mediated deSUMOylation of Drp1.

Drp1 can be modified by both SUMO-1 and SUMO-2/3, resulting in contrasting effects on Drp1 mitochondrial partitioning^18^,^19^,^20^. Therefore, we examined which SUMO paralogue is responsible for regulating the interaction between Drp1 and Mff. We have shown previously that SENP3 removes SUMO-2/3 from Drp1 and, consistent with the reported SUMO-2/3 specificity of SENP3, knockdown of SENP3 does not alter global SUMO-1-ylation (Supplementary Fig. 3). In addition, both SENP2 and SENP5 have been reported to remove SUMO-1 from Drp1^23^,^24^,^25^. We therefore tested if overexpression of SENP2 or SENP5 altered the Drp1-Mff interaction. In stark contrast to the enhanced binding of Drp1 to Mff elicited by SENP3, neither SENP2 nor SENP5 overexpression affected Drp1 binding to Mff (Supplementary Fig. 4a and b). These data confirm that SENP3-mediated deSUMOylation of Drp1 facilitates binding to Mff to recruit Drp1 to mitochondria. In the reciprocal situation our the prediction is that SUMO-2/3-ylation of Drp1 should hinder Drp1 binding to Mff and thereby provide a mechanism to reduce Drp1 partitioning at mitochondria.

### SUMOylation regulates the Drp1-Mff interaction

**Figure 2:**
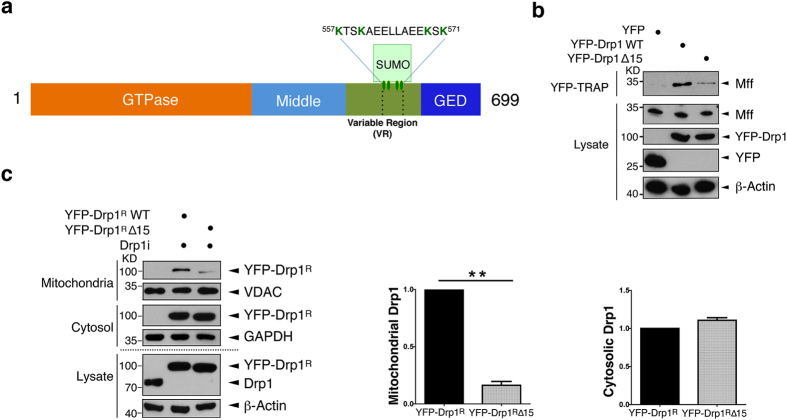
SUMOylation within the VR of Drp1 regulates binding to Mff. (**a**) Schematic of Drp1 domains showing the location of the SUMOylatable lysines within the variable region and the 15 residue sequence removed in Drp1 ∆15. (**b**) YFP-Drp1 ∆15 shows reduced interaction with Mff in HEK293 cells. GFP-TRAP samples and lysates from cells expressing YFP, YFP-Drp1 or YFP-Drp1 ∆15 were immunoblotted with Mff, GFP, and β-actin antibodies. (**c**) Decreased mitochondrial association of YFP-Drp1 ∆15. YFP-Drp1^R^ WT or Drp1^R^ ∆15 was expressed in HEK293 cells after knockdown of endogenous Drp1. Whole cell lysates, cytosolic and mitochondrial fractions were blotted as indicated. The histograms show the relative partitioning of WT and ∆15 Drp1 (n = 3 mitochondrial YFP-Drp1^R^, *P = 0.0016; for cytosolic YFP-Drp1^R^, P = 0.0983; Paired Student’s t-test).

The variable region (VR) of Drp1 is required for mitochondrial localisation^5^,^6^ but the mechanism of recruitment has not been established. We therefore asked if modification of the four SUMOylatable lysines within the VR^16^,^17^ affects Drp1 binding to Mff. We removed the target lysines and immediately adjacent residues by deleting the fifteen amino acid sequence (^557^**K**TS**K**AEELLAE**K**S**K**^571^) that is conserved in all Drp1 isoforms (Fig. 2a). This Drp1Δ15 mutant displayed greatly reduced interaction with Mff (Fig. 2b), indicating that the deleted residues may be directly involved in Drp1 binding to Mff. Consistent with this, Drp1Δ15 associated with the MOM much less than wild-type Drp1 (Fig. 2c). The reduced Mff binding observed with Drp1Δ15 parallels the decreased binding observed when Drp1 SUMOylation is promoted by knocking down SENP3 (Fig. 1d). Taken together, we interpret these results to suggest that the structural attachment of the SUMO peptide to the VR sterically interferes with Drp1 binding to Mff. We note, however, that we cannot definitively rule out that deletion of these 15 amino acids causes misfolding of the VR that could itself disrupt mitochondrial localisation of Drp1.

### Preventing Drp1 binding to Mff is cytoprotective

**Figure 3:**
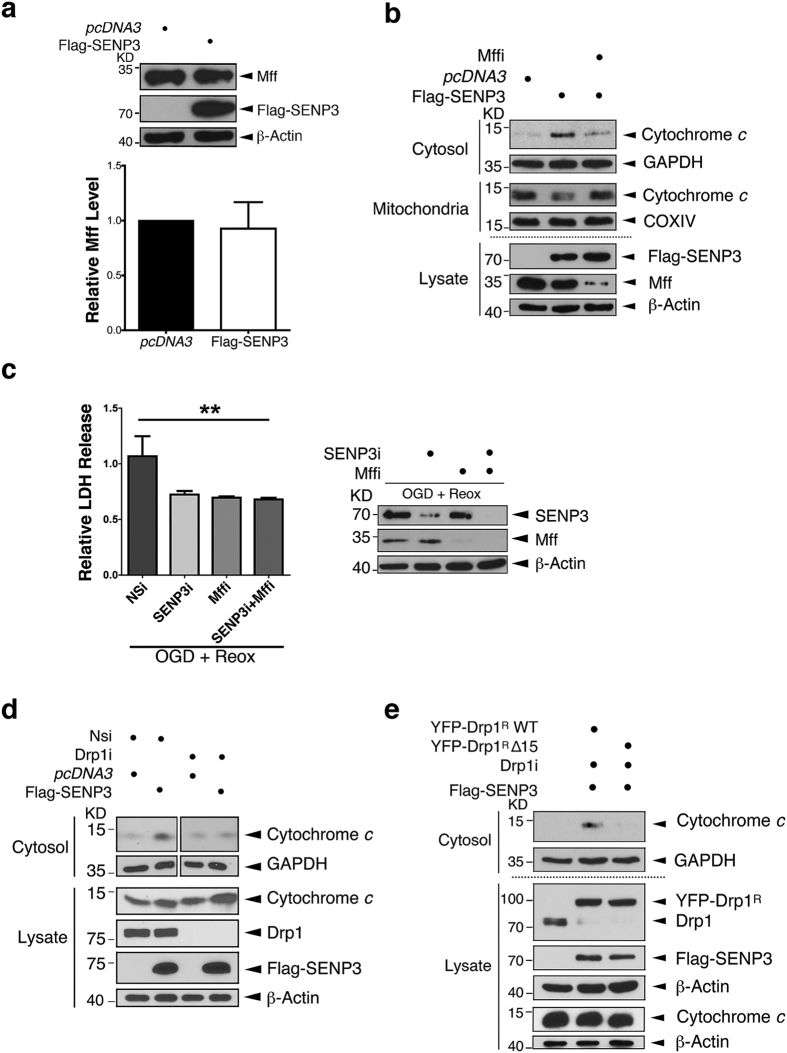
SENP3 regulation of the Drp1-Mff interaction mediates cytochrome *c* release. (**a**) Overexpression of SENP3 does not change the levels of endogenous Mff in HEK293 cells. (n = 3; P = 0.7902; Paired Student’s t-test). (**b**) Knockdown of Mff inhibits SENP3-mediated cytochrome *c* release. Flag-SENP3 was transfected into Mff knockdown HEK293 cells (Mffi). Lysates, cytosolic and mitochondrial fractions were blotted for cytochrome *c*, using GAPDH, COX IV and β-actin as loading controls. (**c**) Combined knockdown of both SENP3 and Mff is not additive on OGD plus reoxygenation-evoked LDH release from HEK293 cells (Upper panel; For Nsi, n = 4; for SENP3i, n = 5; for Mffi, n = 5; for SENP3i + Mffi, n = 6; **P = 0.0085; One-way analysis of variance). Immunoblots confirm the efficient knockdown of SENP3 and/or Mff. (**d**) Knockdown of Drp1 prevents SENP3-mediated cytochrome *c* release. Flag-SENP3 was transfected into Drp1 knockdown HEK293 cells (Drp1i). Lysates and cytosolic fractions were blotted for cytochrome *c*, using GAPDH and β-actin as loading controls. (**e**) Replacement of endogenous Drp1 with YFP-Drp1 ∆15 abolishes SENP3-mediated cytochrome *c* release. Flag-SENP3, together either YFP-Drp1^R^ WT or Drp1^R^ ∆15, were transfected into HEK293 cells after knockdown of endogenous Drp1. The cytosolic fraction and whole cell lysate was blotted for cytochrome *c*, GAPDH, Drp1, Flag, or β-actin.

We next investigated if SUMO modulation of the Drp1-Mff interaction provides a direct mechanistic explanation for SENP3-mediated cytochrome *c* release^17^. First we confirmed that overexpression of SENP3 does not alter levels of Mff, to exclude the possibility that enhanced mitochondrial recruitment of Drp1 could be attributable to increased Mff in the MOM (Fig. 3a). Consistent with SENP3 regulating cytochrome *c* release through the Drp1-Mff pathway, Mff knock down decreased the cytochrome *c* release induced by overexpressing SENP3 (Fig. 3b). Furthermore, Mff knockdown decreased LDH release in cells subjected to OGD plus reoxygenation (Fig. 3c). Importantly, there was no additive protection in cells in which both SENP3 and Mff were ablated, consistent with both proteins acting in the same pathway. In addition, similar to knockdown of Mff, knockdown of Drp1 abolished the ability of SENP3 to promote cytochrome *c* release (Fig. 3d). Finally, we knocked down endogenous Drp1 in HEK293 cells and replaced it with either YFP-Drp1^R^WT or YFP-Drp1^R^Δ15 (molecular replacement with RNAi resistant protein denoted by ^R^) and challenged the cells with SENP3 overexpression. As shown in Fig. 3e, cytochrome *c* release was increased in the cells rescued with the YFP-Drp1^R^ WT but not with YFP-Drp1^R^Δ15, which shows reduced binding to Mff. These results indicate that SENP3 deSUMOylation of Drp1 promotes binding to Mff that, in turn, leads to cytochrome *c* release and apoptosis.

### SUMOylation status of Drp1 does not affect its dimerization

**Figure 4:**
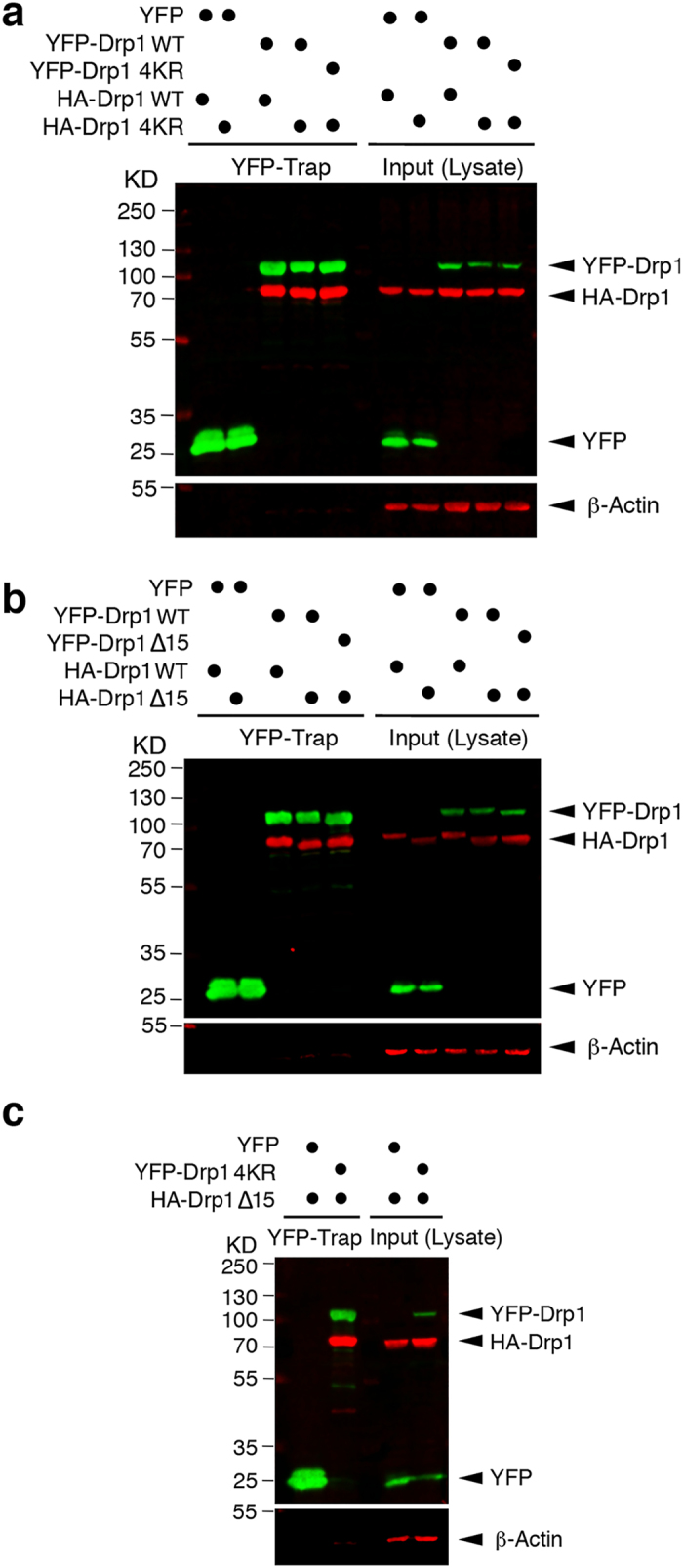
Drp1 SUMOylation does not affect its self-association. (**a**) Ablation of Drp1 SUMOylation does not affect Drp1 dimerization. YFP, YFP-Drp1 WT, or YFP-Drp1 4KR mutant, together with either HA-Drp1 WT or HA-Drp1 4KR mutant, were transfected into HEK293 cells. GFP-TRAP and lysate samples were blotted for HA, GFP, or β-actin as indicated. (**b**) Removal of key lysines for SUMOylation of Drp1 does not affect Drp1 dimerization. YFP, YFP-Drp1 WT, or YFP-Drp1 ∆15 mutant, together with either HA-Drp1 WT or HA-Drp1 ∆15 mutant, were transfected into HEK293 cells. GFP-TRAP and lysate samples were blotted for HA, GFP, or β-actin as indicated. (**c**) Non-SUMOylatable Drp1 is capable of associating with Drp1 without key lysines for SUMOylation. Either YFP or YFP-Drp1 4KR mutant was transfected into HEK293 cells expressing HA-Drp1 ∆15 mutant. GFP-TRAP and lysate samples were blotted for HA, GFP, or β-actin as indicated.

Increasing evidence indicates a significant role for oligomerization in Drp1 mitochondrial recruitment and its function as a GTPase to promote fission. Drp1 dimerization has been shown to be important for Drp1 function^26^,^27^,^28^ and dimerised Drp1 is thought to be the minimal assembly unit for Drp1^29^. To ensure that the effects we observe on Mff binding and mitochondrial association are not mediated through differences in Drp1 assembly, we tested if the SUMOylation status of Drp1 had an effect on its dimerization (self-association). Accordingly, YFP-tagged Drp1, YFP-Drp1 4KR or YFP-Drp1 Δ15 were transfected into HEK293 cells expressing HA-tagged Drp1, HA-Drp1 4KR or HA-Drp1 Δ15. Following YFP trap, the samples were subjected to HA immunoblotting. As shown in Fig. 4, changes in the SUMOylation status of Drp1 do not affect Drp1 dimerization, discounting the possibility that SUMOylation modulates Drp1 mitochondrial localisation and function in fission through altering its oligomerization.

### Drp1 on mitochondria is sufficient to cause cytochrome *c* release

**Figure 5:**
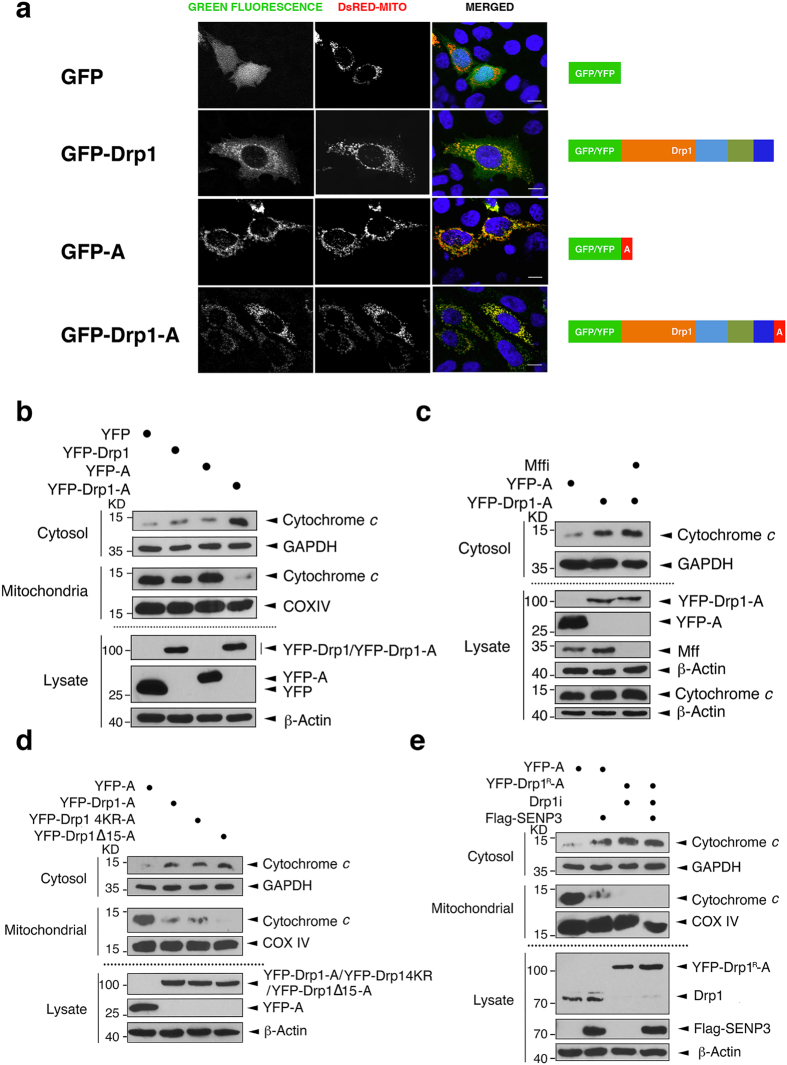
Tethering Drp1 at mitochondria induces cytochrome *c* release independent of Mff. (**a**) GFP-Drp1-A is localized to mitochondria. GFP, GFP-Drp1, GFP-A or GFP-Drp1-A together with DsRed-Mito were transfected into HeLa cells, which were analysed 48 hours post-transfection. Schematics illustrate the constructs GFP/YFP, GFP/YFP-Drp1, GFP/YFP-A and GFP/YFP-Drp1-A. (**b**) YFP-Drp1-A, but not YFP-Drp1, causes cytochrome *c* release. HEK293 cells were transfected as shown and the whole cell lysates, cytosolic and mitochondrial fractions were blotted for cytochrome *c*, GAPDH, COX IV, YFP and β-actin. (**c**) YFP-Drp1-A-induced cytochrome *c* release bypasses Mff. Mff knockdown in HEK293 cells did not prevent cytochrome *c* release mediated by Drp1 directly targeted to the MOM. (**d**) SUMOylation does not affect cytochrome *c* release mediated by Drp1 directly targeted to the MOM. Whole cell lysates, cytosolic and mitochondrial fractions of HEK293 cells expressing YFP-A, YFP-Drp1-A, YFP-Drp1 4KR-A or YFP-Drp1 ∆15-A were blotted for cytochrome *c*, GAPDH, COX IV, YFP, or β-actin. (**e**) Overexpressing SENP3 does not increase cytochrome *c* release in HEK293 cells already expressing YFP-Drp1-A tethered to the MOM. Flag-SENP3, together with either YFP-A or YFP-Drp1^R^-A, were transfected into HEK293 cells after knockdown of endogenous Drp1. The cytosolic fraction or whole cell lysate was blotted for cytochrome *c*, GAPDH, Drp1, Flag, or β-actin.

While these data demonstrate the importance of the Drp1–Mff interaction, they do not address if Mff is required only to recruit Drp1 to the MOM or if Mff plays an integral role in downstream events leading to cytochrome *c* release beyond regulating Drp1 partitioning to the MOM. Therefore, we tested if Drp1 localisation at the MOM without binding to Mff evokes cytochrome *c* release. To do this we fused the mitochondrial-targeting sequence derived from the ActA protein of *Listeria monocytogenes*^30^ to the C-terminus of YFP and YFP-Drp1 (YFP-A and YFP-Drp1-A; Fig. 5a). As expected, both YFP-A and YFP-Drp1-A partition strongly to the mitochondria (Fig. 5a and Supplementary Fig. 5a and b). Overexpression of YFP-Drp1-A (but not YFP-A, YFP, or YFP-Drp1) caused in a marked increase in cytosolic cytochrome *c* under basal conditions (Fig. 5b and Supplementary Fig. 5b). Moreover, consistent with these subcellular fractionation experiments, confocal imaging showed that overexpression of GFP-Drp1-A, but not of GFP-A alone, significantly reduced the mitochondrial localization of cytochrome *c* in HeLa cells (Supplementary Fig. 6). Importantly, knock down of Mff did not prevent Drp1-A evoked cytochrome *c* release (Fig. 5c and Supplementary Fig. 5c). These results confirm that the role of Mff in cytochrome *c* release can be bypassed by tethering Drp1 to mitochondria, indicating that Mff functions by recruiting non-SUMOylated Drp1 to the MOM. Furthermore, overexpressing mitochondrial targeted YFP-Drp1, non-SUMOylatable YFP-Drp1 4KR mutant or YFP-Drp1 Δ15 mutant induces comparable levels of cytochrome *c* release (Fig. 5d and Supplementary Fig. 5d), indicating that neither Drp1 SUMOylation nor Mff binding play roles in cytochrome *c* release beyond regulating mitochondrial targeting of Drp1. Interestingly, however, YFP-Drp1-A did not increase levels of caspase 3 or cleavage of its substrate PARP1 compared to YFP-A (Supplementary Fig. 7). These results suggest that tethering of Drp1 to mitochondria is not, in itself, sufficient to mediate full induction of apoptosis, or that Drp1 promotes cell death through a caspase-independent mechanism, such as autophagic cell death^1^,^31^.

Finally, we asked if SENP3 can further promote cytochrome *c* release in cells where Drp1 has been tethered to the MOM. As shown in Fig. 5e, following knockdown of endogenous Drp1 and replacement with YFP-Drp1^R^-A, overexpression of SENP3 did not release additional cytochrome *c*. While we cannot completely exclude the possibility that SENP3 is ineffective because Drp1-A has fully saturated cytochrome *c* release, we interpret these findings to indicate that SENP3-mediated Drp1 deSUMOylation enhances binding to Mff to recruit Drp1 to the MOM.

## Discussion

**Figure 6:**
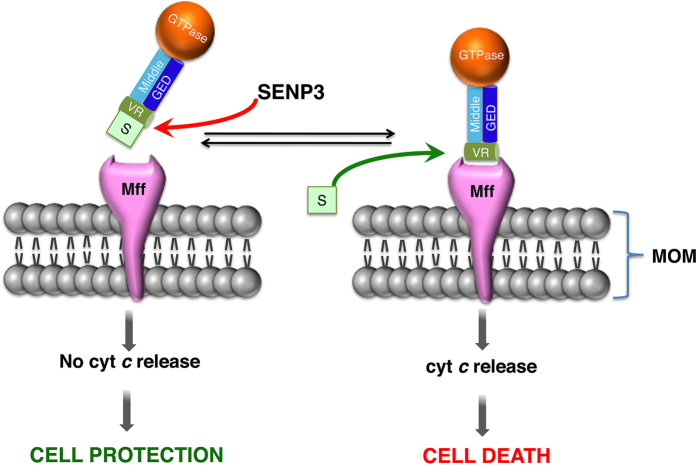
Schematic illustrating how SUMOylation prevents Drp1 interaction with Mff at the MOM. SENP3 deSUMOylates Drp1, increasing the ability of Drp1 to bind Mff, which leads to cytochrome *c* release. S, SUMO-2/3.

Here we provide a novel mechanistic explanation of how SENP3 promotes cell death during reperfusion after ischemia. We show that SENP3 deSUMOylates Drp1, which promotes mitochondrial association via enhanced binding of Drp1 to the mitochondrial adaptor protein Mff, which in turn, leads to cytochrome *c* release (Fig. 6). These results add to the emerging realisation that Drp1 is subject to highly sophisticated post-translational controls, and provide molecular understanding of how SUMO-2/3-ylation controls the partitioning of Drp1 to the mitochondrial membrane. Previous studies have reported that Drp1 phosphorylation by either cyclic AMP-dependent protein kinase A or calcium/calmodulin-dependent protein kinase inhibits mitochondrial recruitment of Drp1 and mitochondrial fission. In contrast, phosphorylation at a different serine by cyclin-dependent kinase 1 enhances Drp1 recruitment and fission, as does S-nitrosylation within the VR domain^3^,^32^,^33^,^34^. However, in most cases, it is unknown how these modifications alter Drp1 mitochondrial association on a molecular level. We have extended these findings by demonstrating that SUMO modification within the Mff binding site on Drp1 reduces binding to Mff, most likely through the attached SUMO occluding the binding site. Indeed, given that CamKIα phosphorylation of Drp1 promotes its mitochondrial localization by increasing its affinity for the receptor Fis1^33^, our results suggest that regulating the interactions of Drp1 with mitochondrial receptors may constitute a general mechanism by which post-translational modifications control the mitochondrial association of Drp1 under both physiological and pathophysiological conditions.

During ischemia, SENP3 levels decrease, promoting Drp1 SUMOylation. However, during reperfusion SENP3 levels recover causing, among other things, deSUMOylation of Drp1 and consequent mitochondrial association and cytochrome *c* release. Here, we demonstrate the SENP3-mediated cytochrome *c* release requires Mff. Importantly, this requirement can be bypassed by tethering Drp1 to mitochondria which occludes SENP3-mediated cytochrome *c* release, independent of the SUMOylation status of Drp1. Thus, SENP3 appears to mediate cytochrome *c* release solely by enhancing the interaction between Mff and Drp1, through deSUMOylating Drp1. In addition, our data suggest that the primary role of Drp1 SUMO-2/3-ylation is to inhibit the Drp1-Mff interaction, and that the role of Mff in SENP3-mediated cytochrome *c* release is to recruit Drp1 to the MOM.

Previous work has demonstrated that both SUMO-1^35^ and SUMO-2/3^17^ can modify Drp1. SUMO-1-ylation of Drp1 has been reported to promote the stable association with MOM during cell stress^18^, although the role of Drp1 SUMO-1-ylation in cell death remains unclear. Conversely, increased SUMO-2/3-ylation of Drp1 reduces recruitment to the MOM^17^. Overexpression of SENP3 to deSUMOylate Drp1, or expression of the non-SUMOylatable Drp1 4KR mutant, enhances Drp1 partitioning to the MOM, increases mitochondrial fission and causes the release of cytochrome *c*^17^. Although many details need to be resolved, current evidence suggests that Drp1 modification by SUMO-1 can promote mitochondrial fission whereas SUMO-2/3 modification prevents it, potentially providing a highly responsive and nuanced SUMO-mediated regulation system to control mitochondrial dynamics. It is noteworthy, however, that our data suggest that blocking all SUMOylation of Drp1 by mutating the SUMO acceptor sites favours mitochondrial association of Drp1, by enhancing its binding to Mff. Moreover, while SENP3 overexpression enhances Drp1 binding to Mff, overexpression of SENP2 or SENP5, which have been reported to remove SUMO-1 from Drp1^23^,^24^,^25^ had no effect, suggesting that modulation of the Drp1-Mff interaction is primarily mediated by SUMO-2/3-ylation of Drp1. Exactly how SUMO-1 and SUMO-2/3 can exert differential effects on Drp1 mitochondrial localization is currently unclear, and further work will be required to reconcile these findings. Nonetheless, our observation that SUMOylation of Drp1 mediates its partitioning to mitochondria during ischemia, through regulation of the Drp1-Mff interaction, demonstrates the importance of this interaction in determining cell survival to extreme stress, and highlights this pathway as a potential molecular target for intervention in the treatment of stroke.

In summary, we show that deSUMOylation of Drp1 by SENP3 enhances its interaction with the mitochondrial adaptor protein Mff, which recruits Drp1 to the MOM to promote cytochrome *c* release and cell death. These data provide new molecular insights into the mechanisms underpinning cell stress responses and demonstrate how, by modulating the dynamic equilibrium of Drp1 recruitment to mitochondria, SENP3 constitutes a crucial arbiter of cell fate during ischemia and reperfusion.

## Methods

### Plasmids and mutagenesis

DNA constructs encoding Flag-SENP3, HA-Drp1 (human isoform 3), YFP-Drp1 (human isoform 3) were described previously^17^. cDNA for the mitochondrial target sequence encoding residues 599–624 of the ActA protein from *Listeria monocytogens* was obtained from pEF6-Bcl-xL-ActA which was provided by C. Li^30^. YFP-A/GFP-A and YFP-Drp1-A/GFP-Drp1-A were generated by insertion of the mitochondrial target sequence by PCR-based mutagenesis. GFP-Mff was provided by G.K. Voeltz^31^. cDNAs encoding the mouse Fis1, MID49, or MID51 were kindly provided by D.C. Chan. GST-constructs were generated by insertion of the relevant cDNA into the Bam H1/Not1 sites of pEBG or pGEX-4T-1.

HA-Drp1 4KR, YFP-Drp1 4KR, and RNAi-resistant YFP-Drp1 (YFP-Drp1^R^) or YFP-Drp1 4KR (YFP-Drp1^R^ 4KR) were prepared as described previously^17^. YFP-Drp1 Δ15 and RNAi-resistant YFP-Drp1 Δ15 (YFP-Drp1^R^Δ15) were made by PCR-based mutagenesis. Mff ΔN50 deletion mutant (residues 51–342) was generated by PCR and subcloned into pEBG.

### Cell Culture

HEK293 cells and HeLa cells were cultured using standard procedures in Dulbecco’s modified Eagle’s medium (DMEM; Lonza) containing 10% foetal bovine serum (FBS), 5 mM glutamine, and 100 units/ml penicillin/streptomycin at 37 °C in humidified air supplemented with 5% CO_2_.

### DNA and siRNA Transfections

DNA, siRNA or DNA & siRNA was transfected into HEK293 cells and HeLa cells using jetPEI, INTERFERin or jetPRIME (Polyplus Transfection), respectively. siRNA duplexes used were as follows: human SENP3 siRNA (Santa Cruz)^17^, human Mff siRNA (Eurofins MWG Operon; target sequences CCAUUGAAGGAACGUCAGA and GCA GAUCUUGACCUUAUUC) and human Drp1 siRNA (Eurofins MWG Operon)^17^.

### Subcellular Fractionation

Cytosolic and mitochondria fractions from HEK293 cells were prepared using a ProteoExtract Cytosol/Mitochondria Fractionation Kit (Calbiochem) as described previously^17^.

### Oxygen-Glucose Deprivation and Reoxygenation

OGD was performed within a MACS-VA500 anaerobic workstation (Don Whitley Scientific Limited) supplemented with 95% N_2_ and 5% CO_2_ at 37 °C. Briefly, HEK293 cells were washed with deoxygenated DMEM without glucose containing 0.1% FBS (Invitrogen) and 5 mM glutamine, and the cells were maintained in the medium under the anaerobic conditions monitored using anaerobic indicator strips (Thermo Fisher Scientific). Control cells had their culture medium removed and were washed, and medium replaced by fresh DMEM containing glucose, 0.1% FBS, and 5 mM glutamine. For reoxygenation experiments HEK293 cells were transferred from the anaerobic workstation and kept at 37 °C in humidified air supplemented with 5% CO_2_.

### LDH Assay

LDH levels in conditioned culture media were assessed using the *In Vitro* Toxicology Assay Kit (Lactic Dehydrogenase Based; Sigma) according the manufacturers instructions. Results shown in each histogram are representative of at least three experiments carried out using different cell populations.

### Preparation of recombinant proteins, cell lysates, immunoprecipitation, GFP-Trap and GST pulldown

His-tagged Drp1 WT or 4KR mutant were produced from the vector pET21a using BL21(DE3) *E. coli* as described previously^32^,^33^. Whole cell lysates from transfected HEK293 cells were prepared as previously described^17^. GFP or YFP-tagged proteins were isolated by incubating lysates with GFP-Traps_A beads (ChromoTek). Lysates were incubated with glutathione-Sepharose 4B (Generon) to isolate GST-tagged proteins. In experiments to immunoprecipate endogenous SUMO-2/3 conjugates from whole cell lysates using SUMO-2/3 antibody (Clone 1E7, MBL), cells were lysed in a buffer containing 20 mM Tris, pH 7.4, 137 mM NaCl, 25 mM β-glycerophosphate, 2 mM sodium pyrophosphate, 2 mM EDTA, 1% Triton X-100, 10% glycerol, 1× protease inhibitor cocktail (Roche) plus 1% SDS, and boiled for 10 min. The cell lysates were then diluted 10 folds in the same buffer without SDS in the presence of 20 mM N-Ethylmaleimide (NEM). In experiments to detect endogenous interaction between Mff and Drp1 by immunoprecipitation using Mff antibody (Proteintech), cell lysates were prepared as described previously for Bcl-2 complex detection^34^ with some modifications. Briefly, HEK293 cells were washed once with PBS, and then fixed with 0.4% PFA in PBS at room temperature. 10 min later 120 mM Glycine was added to quench the PFA. Fixed cells were then spun down, and the cell pellet was lysed in a buffer containing 20 mM Tris, pH 7.4, 137 mM NaCl, 25 mM β-glycerophosphate, 2 mM sodium pyrophosphate, 2 mM EDTA, 1% Triton X-100, 10% glycerol, 1× protease inhibitor cocktail (Roche). To precipitate endogenous Mff, the cell lysate was incubated overnight with the Mff antibody pre-conjugated to protein-A beads (Sigma). The protein-A beads were spun down, washed and resuspended in SDS sample buffer for immunoblotting. To detect the interaction between Drp1 and Mff *in vitro*, His-tagged Drp1 WT or 4KR mutant was mixed with GST or GST-Mff, fixed with 0.4% PFA in PBS at room temperature and quenched 10 min later with 120 mM glycine. Following GST-pulldown the pellet was spun down, washed and resuspended in SDS sample buffer for immunoblotting.

### Immunoblotting

Samples were resolved by SDS-PAGE (7.5–15% gels), transferred to Immobilon-P membranes (Millipore Inc.) and immunoblotted as indicated. Primary antibodies were used to detect Cox IV (3E11, Cell Signaling), cytochrome c (D18C7, Cell Signaling), Drp1 (BD Biosciences; D6C7, Cell Signaling; H-300, Santa Cruz biotechnology), Flag (Sigma), GAPDH (Abcam), GFP (FL, Santa Cruz biotechnology; Roche), GST (GE Healthcare), HA (Sigma), Mff (Proteintech; Sigma), RhoGDI (Abcam), SENP3 (D20A10, Cell Signaling), and β-actin (Sigma). Immune complexes were detected either using HRP-conjugated secondary antibodies (Sigma) followed by enhanced chemiluminescence (Thermo Scientific Pierce) or using fluorescent secondary antibodies (LI-COR). Each immunoblot presented is representative of at least three experiments carried out using different cell populations.

### Cell Imaging Assay

Cells were fixed for 12 min at room temperature (RT) in 4% paraformladehyde/PBS with 5% sucrose. Cell imaging assay was performed as previously described^17^.

### Statistics

All statistical analyses were performed using either paired/unpaired Student’s test with two-tail P-value or one-way analysis of variance followed by Bonferroni post-test as appropriate. Value are presented as mean ± SEM and are normalised to the control value.

## Additional Information

**How to cite this article**: Guo, C. *et al*. SENP3-mediated deSUMOylation of Drp1 facilitates interaction with Mff to promote cell death. *Sci. Rep.* **7**, 43811; doi: 10.1038/srep43811 (2017).

**Publisher's note:** Springer Nature remains neutral with regard to jurisdictional claims in published maps and institutional affiliations.

## Supplementary Material

Supplementary Figures
