# Supplementary material for: SENP3-mediated deSUMOylation of Drp1 facilitates interaction with Mff to promote cell death
Source: Sci Rep. 2017 Mar 6;7:43811. doi: 10.1038/srep43811 (PMC5338345; doi:10.1038/srep43811)
Supplement: Supplementary Figures [file srep43811-s1.doc]

**SUPPLEMENTARY ONLINE MATERIAL**

**SENP3-mediated deSUMOylation of Drp1 facilitates interaction with Mff to promote cell death**

Chun Guo*^§¶^, Kevin A Wilkinson^¶^, Ashley J. Evans, Philip P Rubin, and Jeremy M Henley*

School of Biochemistry, Centre for Synaptic Plasticity, Biomedical Sciences Building, University of Bristol, University Walk, Bristol, BS8 1TD, U.K.

^¶^ Co-first authors

^§^ Present address: Department of Biomedical Science, University of Sheffield, Firth Court, Western Bank, Sheffield, S10 2TN, U.K.

*Please address correspondence to either: [c.guo@sheffield.ac.uk](mailto:c.guo@sheffield.ac.uk) or [j.m.henley@bristol.ac.uk](mailto:j.m.henley@bristol.ac.uk)

This supplement contains:

7 Supplementary Figures

**
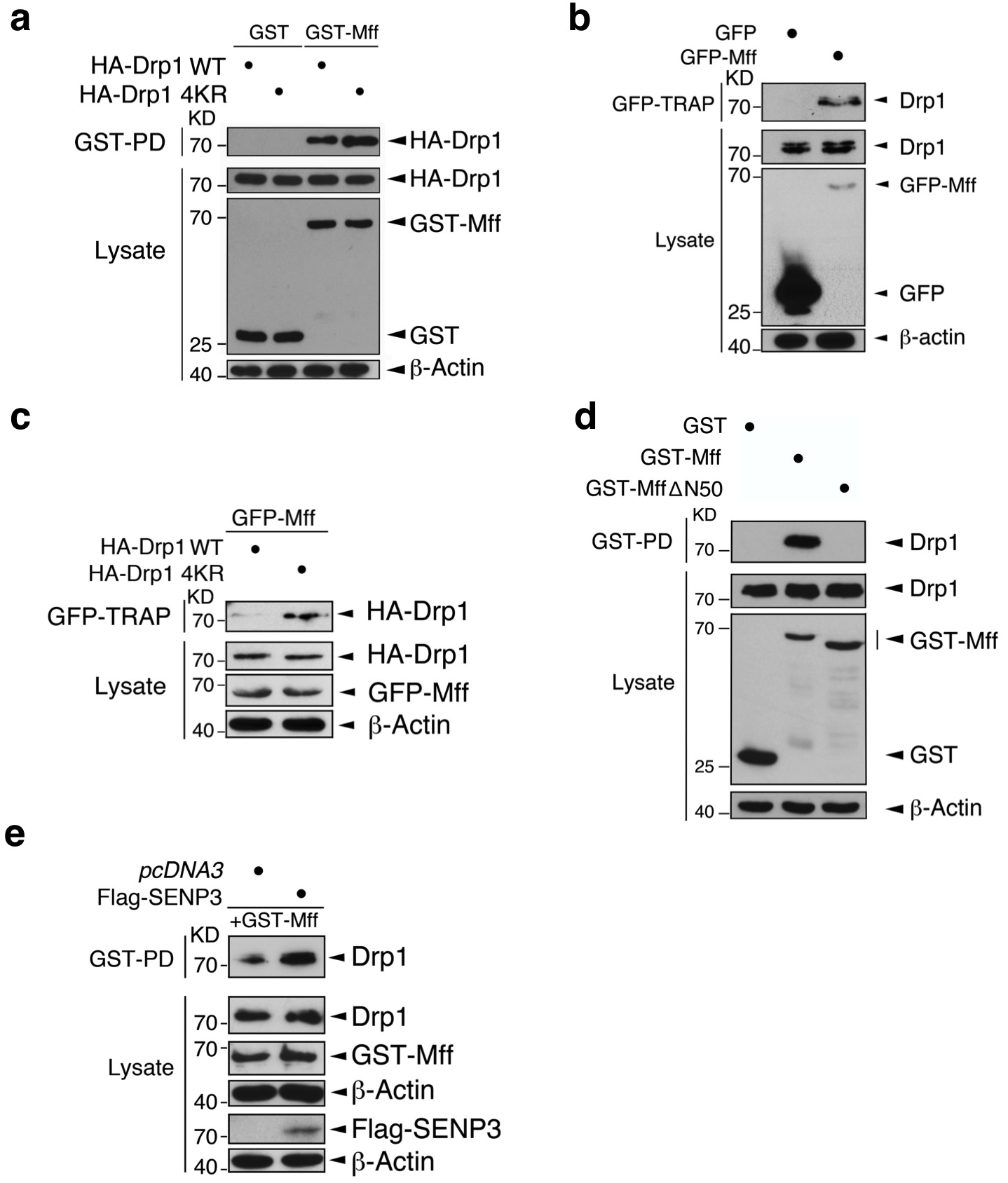
**

**Supplementary Figure 1** DeSUMOylation of Drp1 promotes its binding to Mff.

(**a**) Non-SUMOylatable HA-Drp1 shows enhanced association with GST-Mff in HEK293 cells. Full blots of data shown in **Fig. 1a**. (**b**) Non-SUMOylatable HA-Drp1 shows enhanced association with GFP-Mff in HEK293 cells. GFP-Mff was pulled down using GFP-TRAP and blotted as shown. (**c**) Overexpressed GFP-Mff interacts with endogenous Drp1. GFP-TRAP pulldowns were blotted as shown. (**d**) GST**-**Mff ∆N50 does not bind Drp1 in HEK293 cells. GST-pulldowns were blotted as shown. (**e**) Overexpression of SENP3 increases the Mff-Drp1 interaction. Flag-SENP3 was transfected into HEK293 cells expressing GST-Mff. GST-pulldown and lysate samples were immunoblotted as shown.

**
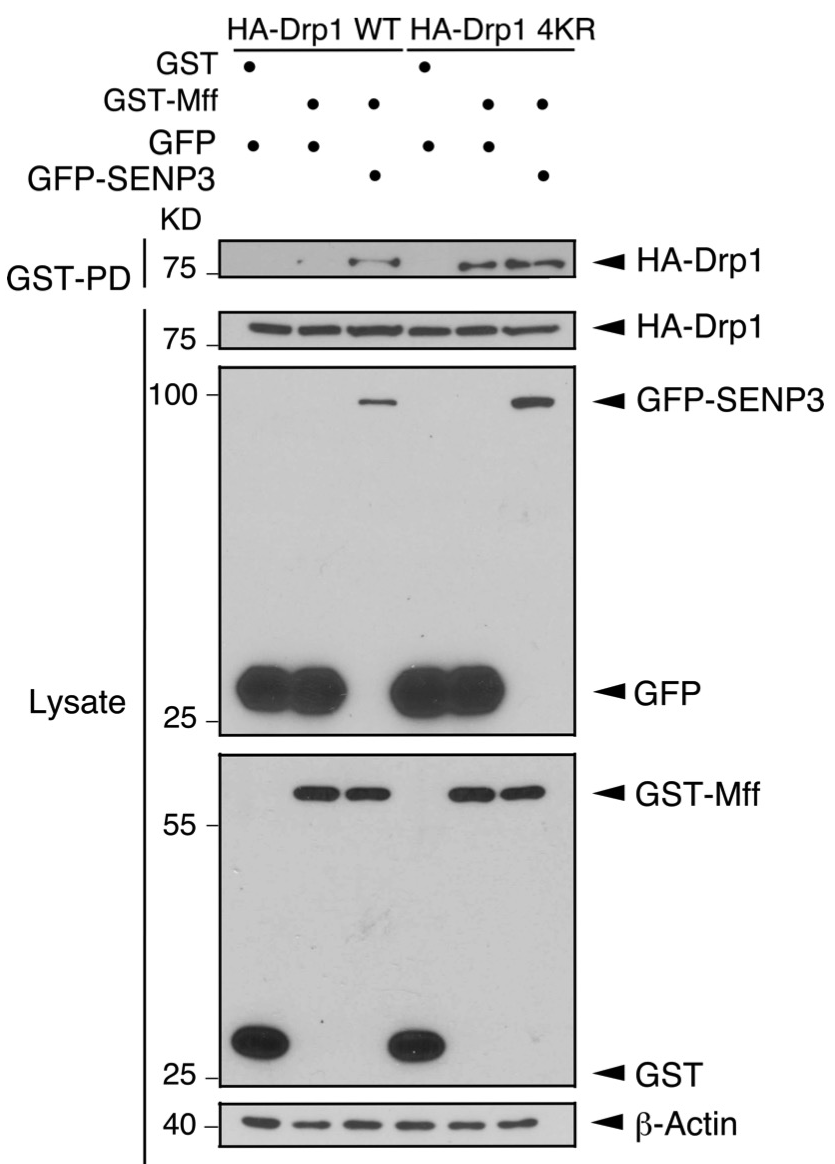
**

**Supplementary Figure 2** SENP3 promotes binding of Drp1 but not its non-SUMOylatable mutant to Mff.

GFP-SENP3 was transfected into HEK293 cells expressing GST-Mff and HA-Drp1 WT/HA-Drp1 4KR. GST-pulldown and lysate samples were immunoblotted as shown.


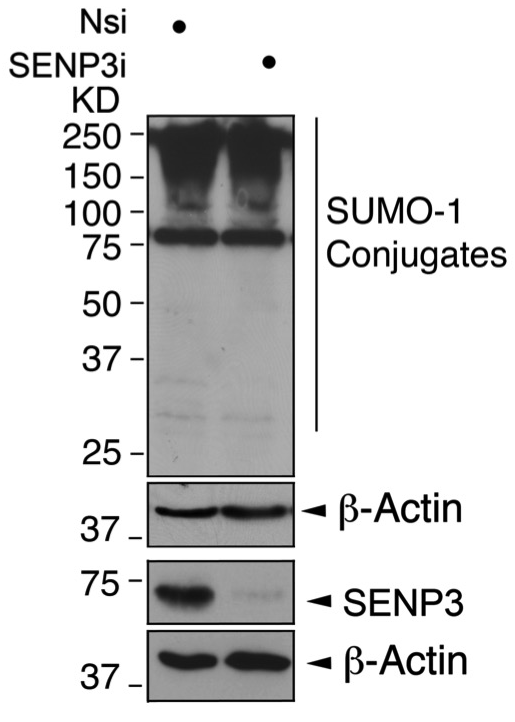


**Supplementary Figure 3** SENP3 knockdown does not change global SUMO-1-ylation.

Non-specific or SENP3 knockdown constructs (Nsi or SENP3i) were transfected into HEK293 cells. Lysate samples were immunoblotted as shown.


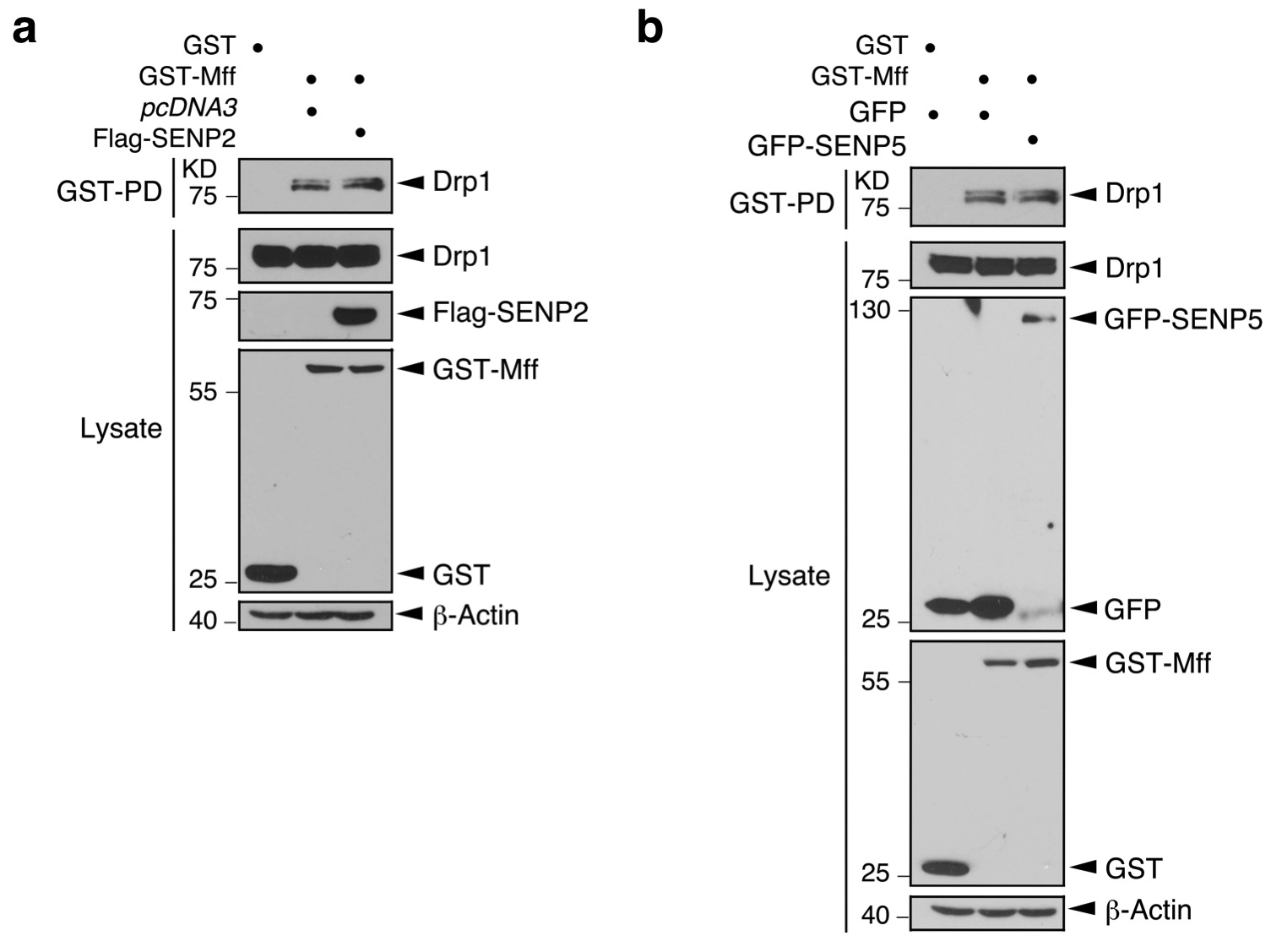


**Supplementary Figure 4** Neither SENP2 nor SENP5 promotes Drp1 binding to Mff.

Flag-SENP2 or GFP-SENP5 were transfected into HEK293 cells expressing GST-Mff. GST-pulldown and lysate samples were immunoblotted as shown.

**
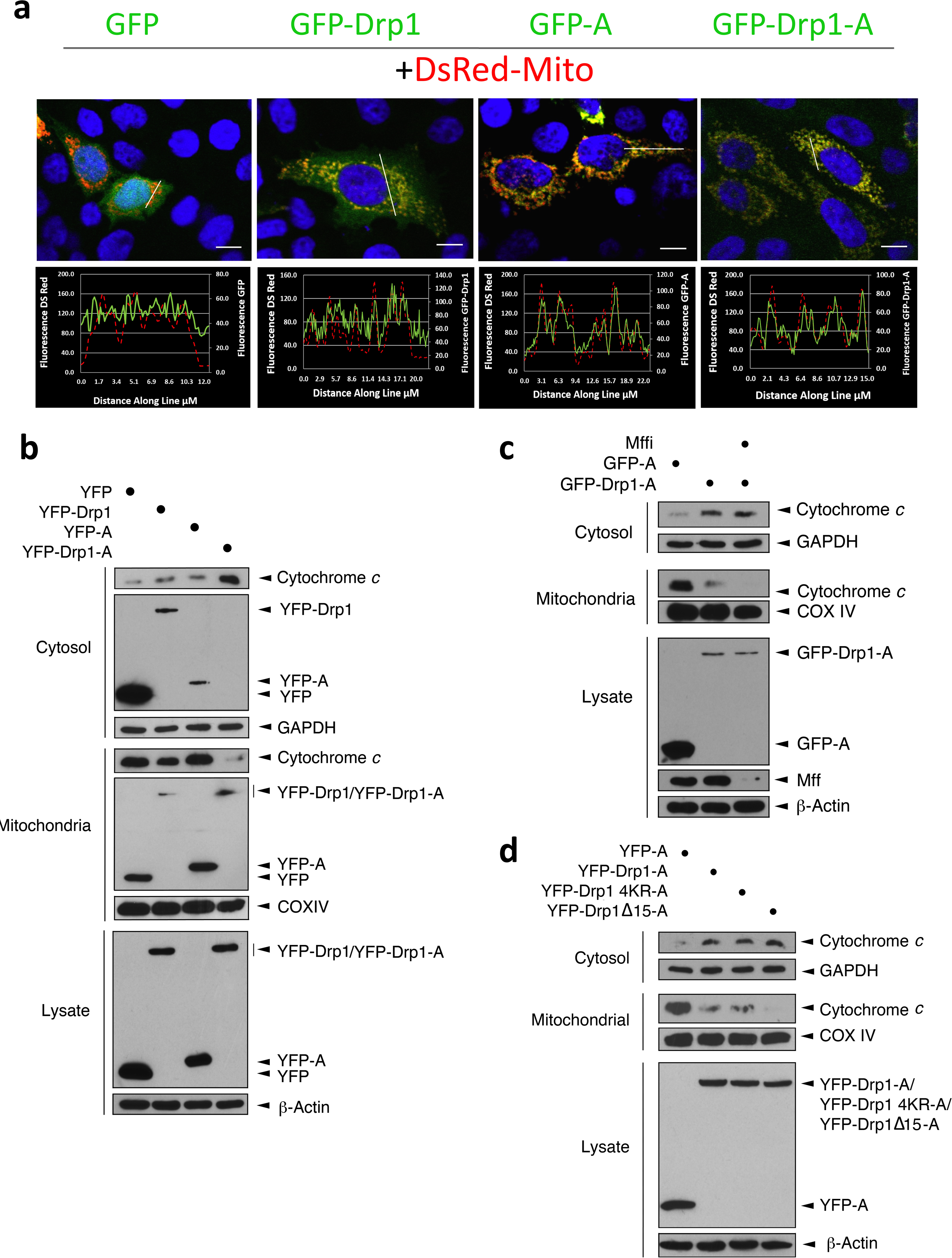
**

**Supplementary Figure 5** Expressing Drp1-A induces Cytochrome *c* release in HEK293 cells.

(**a**) Co-localization analysis of GFP, GFP-Drp1, GFP-A, or GFP-Drp1-A with DsRed-Mito (further details for **Fig.** **5a**). (**b**) Subcellular distribution of YFP, YFP-Drp1, YFP-A, or YFP-Drp1-A in HEK293 cells (complete version of **Fig.** **5b**). (**c**) GFP-Drp1-A-induced cytochrome *c* release bypasses Mff. (**d**) Tethering Drp1 at mitochondria induces cytochrome *c* release independent of the SUMOylation status of Drp1 (complete version of **Fig.** **5d**).

**
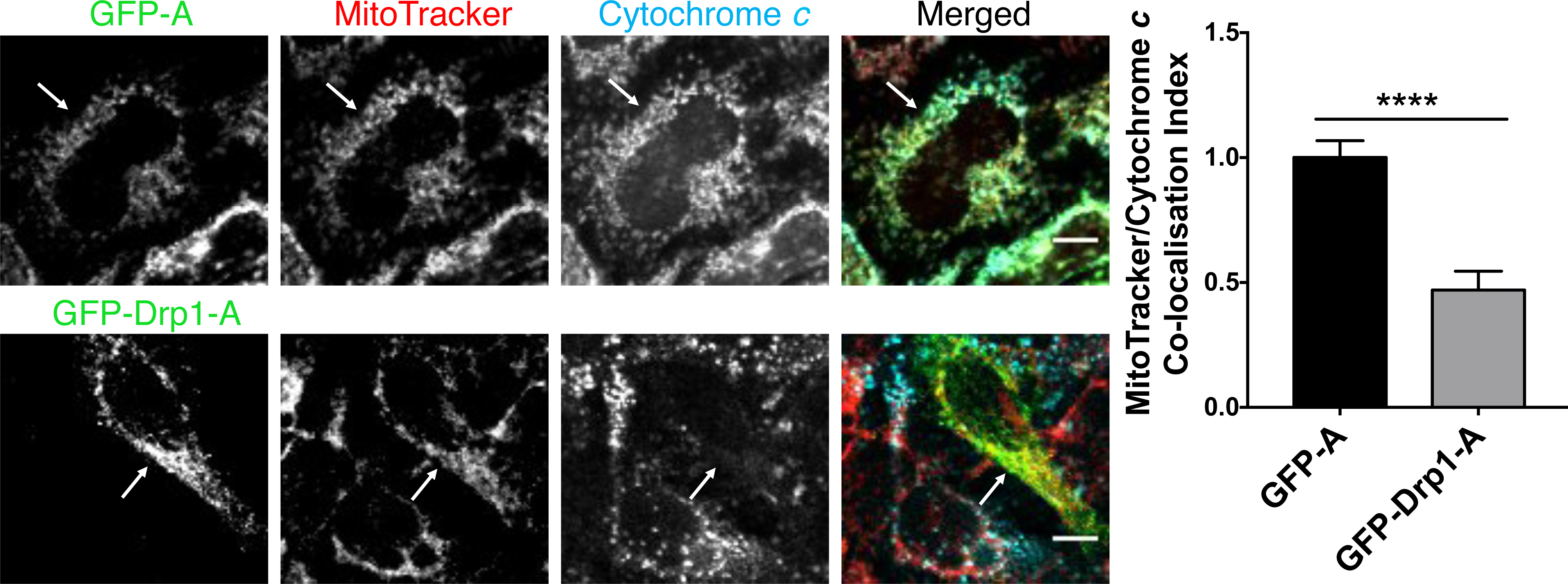
**

**Supplementary Figure 6** Expression of GFP-Drp1-A decreases mitochondrial cytochrome *c* in HeLa cells.

GFP-A or GFP-Drp1-A was transfected into HeLa cells. Following fixation, cells were labelled with MitoTracker and immunostained to assess the mitochondrial localization of cytochrome ***c***. White arrows (Left panel) indicate GFP‐A or GFP‐Drp1-A expressing cells (Green: GFP-A or GFP-Drp1-A; Red: MitoTracker; Soft Cyan: cytochrome ***c***; scale bar: 10 μm). The histogram (Right panel) shows lower levels of the mitochondrial cytochrome *c* in cells expressing GFP-Drp1-A than in cells expressing GFP-A (n=40 cells for GFP‐A and n=40 for GFP‐Drp1-A from 3 independent experiments using different cell populations; **** p=1.2632E-06; Unpaired Student's test).

**
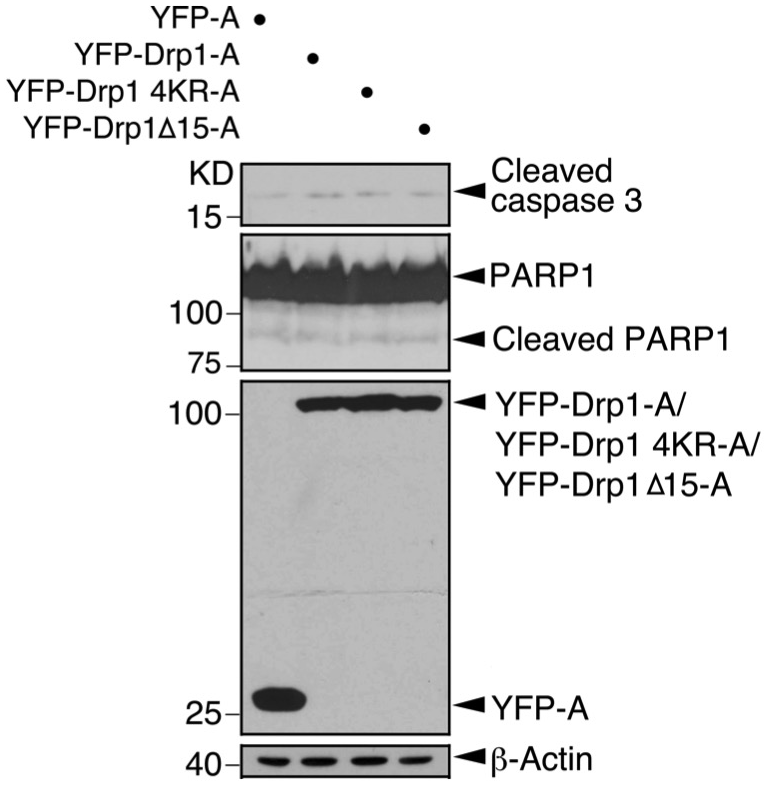
**

**Supplementary Figure 7** Drp1-A expression does not cause cleavage of either caspase 3 or PARP1.

YFP-A, YFP-Drp1-A, YFP-Drp1 4KR-A or YFP-Drp1 Δ15-A were transfected into HEK293 cells. Lysate samples were immunoblotted as shown.
